# Childhood conscientiousness predicts the social gradient of smoking in adulthood: a life course analysis

**DOI:** 10.1136/jech-2014-204263

**Published:** 2015-04

**Authors:** Michael Pluess, Mel Bartley

**Affiliations:** 1 Department of Biological and Experimental Psychology, School of Biological and Chemical Sciences, Queen Mary University of London, London, UK; 2 Department of Epidemiology and Public Health, University College London, London, UK

**Keywords:** SMOKING, Cohort studies, Life course epidemiology, PSYCHOLOGY, SOCIAL INEQUALITIES

## Abstract

**Background and aims:**

The social gradient in smoking is well known, with higher rates among those in less advantaged socioeconomic position. Some recent research has reported that personality characteristics partly explain this gradient. However, the majority of existing work is limited by cross-sectional designs unsuitable to determine whether differences in conscientiousness are a predictor or a product of social inequalities. Adopting a life course perspective, we investigated in the current paper the influence of conscientiousness in early and mid-life on the social gradient in smoking and the role of potential confounding factors in a large longitudinal cohort study.

**Methods:**

Using data from the 1958 National Child Development Study, we examined the extent to which two measures of conscientiousness, one assessed with a personality questionnaire at age 50 and one derived from three related items at 16 years in childhood, explained the social gradient of smoking at age 50 by comparing nested logistic regression models that included social class at birth, cognitive ability, attention and conduct problems at age 7, and educational qualification.

**Results:**

Childhood conscientiousness was a significant predictor of smoking at 50 years (OR=0.86, CI (95%) 0.84 to 0.88), explaining 5.0% of the social gradient independent of all other variables. Childhood conscientiousness was a stronger predictor than adult conscientiousness, statistically accounting for the observed direct association of adult conscientiousness with smoking.

**Conclusions:**

Conscientiousness may be a predictor rather than a product of social differences in smoking. Inclusion of personality measures and adoption of a life course perspective add significantly to our understanding of health inequalities.

## Introduction

Empirical studies consistently demonstrate the existence of a social gradient in health outcomes, with quality of health generally declining from the most to the least advantaged socioeconomic groups, whether these be defined in terms of income, education, social status or conditions of employment.^1–3^ There is some debate as to the extent to which such social inequalities in health are explained by health-risk behaviours such as diet, exercise and smoking,^4^ ^5^ with recent work suggesting that behaviour may be a more significant factor than previously thought.^6^ ^7^ Of the behavioural risk factors, many studies indicate that smoking is the most important.^3^ The social distribution of smoking is well known, with higher rates among social groups with lower levels of education and income in English-speaking and Northern European nations. However, merely identifying the importance of health-risk behaviours takes us only one step closer towards understanding health inequality.^8^ In fact, decades of health education have failed to narrow social disparities in health.

Some recent literature has explored associations between health-risk behaviours and personality characteristics, providing first empirical evidence for the hypothesis that inequalities in health behaviours may also be influenced by the social distribution of personality traits. For example, type A behaviour pattern—a personality trait associated with impulsivity and aggression—explained over 28% of the association between education and smoking in a sample of 477 young Finnish men^9^ and reduced the relationship between educational attainment and mortality risk at age 49 by 34%, and the association between income and mortality risk by 28% in a large cohort study.^10^ Recently, the ‘Big Five’ personality traits have been found to explain 20% of the social gradient in mortality.^11^ One of the personality traits that appears to play a predominant role in health outcomes is conscientiousness, which describes the tendency of an individual to be self-controlled, dutiful, reliable and achievement oriented.^12^ Conscientiousness has been found to predict life expectancy,^13–15^ and a systematic review of 194 studies confirms the significant role of conscientiousness as well in health behaviours^16^—including smoking^17^—suggesting that the effects of conscientiousness on mortality may be mediated through health behaviours.^18^

As some have rightly pointed out, cross-sectional analyses do not allow us to assert whether personality traits are a cause or rather a consequence of social experiences.^19^ ^20^ Consequently, attention in studies focused on the role of personality in health inequalities has turned towards the longitudinal prospective investigation of personality across the life course.^21^ ^22^ Initial evidence from such longitudinal studies confirms conscientiousness as an important predictor rather than a consequence of health inequalities. For example, higher conscientiousness in childhood has been found to predict less smoking and better self-rated health 40 years later.^23^ As suggested by Lodi-Smith *et al*,^24^ this positive health effect of conscientiousness may be mediated by educational attainment and health-related behaviours. However, some cross-sectional empirical work suggests that education and conscientiousness are independently associated with smoking,^17^ consistent with findings from a prospective study in which childhood conscientiousness was found to have significant and direct protective effects on adult smoking behaviour after controlling for family socioeconomic status.^25^ Recently, Moffitt *et al*^26^ were able to show that self-control—a central component of conscientiousness—assessed in childhood predicted physical health, substance dependence, personal finance and criminal offending outcomes in adulthood, even when controlling for both intelligence and social class. In summary, there is empirical evidence suggesting that childhood conscientiousness predicts adult health-risk behaviours both directly as well as indirectly (ie, mediated by socioeconomic differences).

The stability of personality across development––and therefore the stage of life at which it is possible to measure personality traits reliably––has been a long-standing matter of debate.^27^ ^28^ The assessment of personality effects across the life course is further complicated by the fact that ‘gold standard’ measures for the ‘Big Five’ personality traits (ie, extraversion, neuroticism, conscientiousness, agreeableness, openness) have only been available for the past 30–40 years. Consequently, personality measures are often not included in older cohorts, or at least not at younger ages of the participants. Even though it has been possible in some cases to create personality measures that reflect some dimensions of the five-factor model in retrospect by using parent or teacher reports of child behaviour,^21^ studies that have investigated relationships between measured personality in childhood and health outcomes in later life are rare.^23^ ^29^

In this paper we set out to combine a concern to explain the stubborn social gradients in health-related behaviour with an interest in the emergence of personality across the life course. We focus on smoking at age 50 years, as the health behaviour with the greatest relative impact on life expectancy with a strong social gradient. Using prospective data from a large British cohort study, we applied nested logistic models in order to investigate the influence of conscientiousness—measured in childhood as well as adulthood—on the social gradient in smoking, taking into account the possible confounding and mediating roles of social class at birth, cognitive ability, attention and conduct problems at age 7, as well as educational qualifications attained across adulthood.

## Methods

Data are taken from the 1958 National Child Development Study (NCDS).^30^ The NCDS is a continuing, multidisciplinary, longitudinal British birth cohort study. It began when data were collected on 18 558 babies born in Great Britain (England, Scotland and Wales) in 1 week in 1958. To date, follow-ups were undertaken when the cohort members were aged 7, 11, 16, 23, 33, 42, 46 and 50 years. Over the years, information has been gathered from a number of sources (ie, parents, schools, cohort members, doctors, medical records). The data included in the current analysis are taken from the 7, 11, 16, 33, 42, 46 and 50 years assessments. (Detailed information on ethics approval and informed consent across the different data collection waves is available.^31^)

### Participants

At the 50 years sweep, only 9790 (52.8%) of the original 18 558 members of the birth cohort provided data: 6.7% had died, 7.4% had emigrated, 6.5% refused and 26.6% could not be contacted. Only 5419 of the 9790 cohort members had complete data. Consequently, all missing data except smoking status at 50 years were imputed applying multiple imputation which resulted in a final sample of 8218. Importantly, cohort members excluded, relative to those included in the final sample, had significantly lower conscientiousness scores in childhood (9.88 vs 10.58), more conduct problems at age 7 (0.23 vs −0.28), lower scores in cognitive ability at age 7 (23.30 vs 24.52), more attention problems at 7 and were more likely to be categorised as a member of a less advantaged social class at birth.

Mean values and SD, as well as frequencies of all included variables of the imputed data, are displayed in table 1 for the total sample as well as separately for smokers and non-smokers.

**Table 1:** Distribution of variables in total sample and according to smoking status at age 50 (N=8218)

Variable	Total sample (N=8218)	Smokers (n=1677, 20.4%)	Non-smokers (n=6541, 79.6%)	Difference between smokers and non-smokers
Sex				
Male	4221 (51.4%)	851 (50.7%)	3370 (51.5%)	
Female	3997 (48.6%)	826 (49.3%)	3171 (48.5%)	p=0.58
Social class at birth				
I	402 (4.9%)	52 (3.1%)	350 (5.4%)	
II	1182 (14.4%)	175 (10.4%)	1007 (15.4%)	
IIIN	842 (10.3%)	146 (8.7%)	696 (10.6%)	
IIIM	3900 (47.4%)	810 (48.3%)	3090 (47.2%)	
IV	911 (11.1%)	202 (12.1%)	709 (10.8%)	
V	981 (11.9%)	292 (17.4%)	689 (10.6%)	p<0.001
Cognitive ability at 7 (1/5ths ‘draw-a-man’)				
Lowest	1641 (20.0%)	383 (22.8%)	1258 (19.2%)	
2nd	1675 (20.4%)	376 (22.4%)	1299 (19.9%)	
Middle	1732 (21.1%)	338 (20.2%)	1394 (21.3%)	
4th	1501 (18.2%)	292 (17.4%)	1209 (18.5%)	
Highest	1669 (20.3%)	288 (17.2%)	1381 (21.1%)	p<0.001
Childhood conduct at 7				
Mean (SD)	−0.28 (2.14)	0.10 (2.38)	−0.38 (2.06)	p<0.001
Minimum/maximum	−7.68/17.57	−5.60/17.57	−7.68/13.87	
Attention problems at 7				
Never	5723 (69.6%)	1115 (66.5%)	4608 (70.5%)	
Sometimes	1979 (24.1%)	433 (25.8%)	1545 (23.6%)	
Frequently	516 (6.3%)	129 (7.7%)	388 (5.9%)	p<0.001
Childhood conscientiousness				
Mean (SD)	10.58 (2.81)	9.39 (2.79)	10.88 (2.73)	p<0.001
Minimum/maximum	3.00/15.00	3.00/15.00	3.00/15.00	
Social class at 50				
I	510 (6.2%)	50 (3.0%)	460 (7.0%)	
II	3382 (41.2%)	541 (32.3%)	2841 (43.4%)	
IIIN	1634 (19.9%)	313 (18.7%)	1321 (20.2%)	
IIIM	1554 (18.9%)	420 (25.0%)	1134 (17.3%)	
IV	907 (11.0%)	273 (16.3%)	634 (9.7%)	
V	231 (2.8%)	80 (4.8%)	151 (2.3%)	p<0.001
Qualifications at 50 (NVQ level)				
None	691 (8.4%)	256 (15.3%)	435 (6.7%)	
1	934 (11.4%)	267 (15.9%)	667 (10.2%)	
2	2144 (26.1%)	468 (27.9%)	1676 (25.6%)	
3	1451 (17.7%)	275 (16.4%)	1176 (18.0%)	
4	2657 (32.3%)	374 (22.3%)	2283 (34.9%)	
5–6	341 (4.1%)	37 (2.2%)	304 (4.6%)	p<0.001
Conscientiousness at 50				
Mean (SD)	33.87 (5.34)	33.33 (5.47)	34.00 (5.29)	p<0.001
Minimum/maximum	11.00/50.00	15.00/48.00	11.00/50.00	

NVQ, National Vocational Qualifications.

### Measures

#### Social class at birth

Childhood social class was based on the mother's husband's occupation at the time of birth, categorised according to the Registrar-General's schema (see ref.[Bibr R32] 32) where ‘I=highest’ and ‘V=lowest’ social class. Social classes were defined as I=professional occupations, II=managerial and technical occupations, IIIN=non-manual skilled occupations, IIIM=manual skilled occupations, IV=partly-skilled occupations and V=unskilled occupations/unemployed/no husband. The Registrar-General's class schema has been variously defined as indicating ‘general standing in the community’ (social status) and ‘occupational skill’ (average qualification level necessary for a given occupation).

#### Cognitive ability at 7

Intelligence at age 7 was measured with the Goodenough-Harris drawing test^33^ in which children are asked to draw a human figure in pencil on a single sheet of paper. Given its non-verbal nature, the ‘draw-a-man’ test is judged to be less influenced by previous academic and cultural experiences, including characteristics of the parental home.^34^

#### Attention problems at 7

Mothers reported at age 7 whether their child has difficulties concentrating on a three-point scale with ‘1=never’, ‘2=sometimes’, ‘3=frequently’.

#### Conduct problems at 7

A scale for conduct problems was created based on three items selected from the teacher-rated Bristol Social-Adjustment Guides (BSAG)^35^ at age 7 years including items ‘hostility towards adults’, ‘Writing off adults & standards’ and ‘Inconsequential behaviour’. All three items were coded so that higher values indicated higher conduct problems, standardised and summed up.

#### Childhood conscientiousness

A measure for conscientiousness in childhood was created based on three childhood items that are conceptually associated with conscientiousness (ie, items related to work and homework). All three items were based on self-report and assessed at age 16. For the first item, participants had to rate themselves on a continuum ranging from ‘1=Lazy’ to ‘5=Hardworking’. The other two items (“I get on with classwork” and “I never take work seriously (reverse-coded)”) were rated on a five-point Likert scale with ‘1=very true’ and ‘5=Not true at all’. All items were coded so that higher values indicated higher conscientiousness, then summed up. According to a factor analysis, all three items loaded on the same factor with factor loadings of each item >0.7, suggesting that the three items reflect the same latent trait of conscientiousness.

#### Social class at 50

Adulthood social class was taken from the interview at age 50, measured according to the Registrar-General's schema with ‘I=highest’ and ‘V=lowest’ social class (see Social Class at Birth for more details).

#### Qualifications at 50

Highest level of an academic or vocational qualification attained by age 50 was derived from measures at ages 33, 42 and 46 years according to the National Vocational Qualifications (NVQ) schema and rated on a six-point scale ranging from ‘1=no qualifications’ to ‘6=degree level or higher’.

#### Conscientiousness at 50

At age 50, cohort members were administered a ‘Big Five’ personality traits questionnaire using 50 items from the International Personality Item Pool (IPIP).^36^ Only conscientiousness was included in the current analysis, and was based on 10 statements (eg, “I am always prepared”, “I pay attention to details”) rated by cohort members on a five-point scale ranging from ‘1=very inaccurate’ to ‘5=very accurate’. Internal consistency in the final sample was acceptable with α=0.77.

#### Smoking at 50

At age 50 years, cohort members were asked to report whether they “smoke cigarettes every day” (n=1401), “smoke cigarettes occasionally but not every day” (n=276), “used to smoke cigarettes but don't at all now” (n=2594) or “never smoked cigarettes” (n=3947). For the purpose of this analysis, smoking at age 50 was dichotomised for use as dependent variable with “Yes=smokes occasionally or every day (n=1677)” and “No=does not smoke at all (n=6541)”.

### Data analysis

Nested logistic regression models^37^ ^38^ were compared to investigate the influence of adult and childhood conscientiousness on the social gradient of smoking at age 50 while controlling for the effects of adulthood and childhood covariates. The uncorrected social gradient for smoking based on social class at age 50 was tested in an initial model 0 (controlled for sex). Models 1–7 then successively introduced adult conscientiousness, educational qualifications at 50, cognitive ability at age 7, attention problems at age 7, conduct problems at age 7, social class at birth and childhood conscientiousness. In more detail, model 1 included the adult measure of conscientiousness to test the extent to which the social gradient of smoking is explained (ie, reduced) by conscientiousness at 50. In model 2, educational qualifications attained up to age 50 were further added in order to test whether effects of adult conscientiousness on the social gradient of smoking were independent of educational attainment. Model 3 introduced a measure of cognitive ability in childhood, based on work suggesting associations between adult conscientiousness and cognitive measures as well as between childhood intelligence and smoking in adulthood. Model 4 included attention problems at age 7 in order to control for the possibility that children's ability to focus their attention may confound effects of conscientiousness and model 5 introduced a measure of conduct problems at age 7 based on recent findings that conduct problems mediated the effects of childhood self-control on adult outcomes.^39^ Model 6 took into account the possibility that childhood social class may be the common explanation of the relationship of all other variables to each other and to smoking. The final model 7 introduced the derived measure of childhood conscientiousness to investigate whether conscientiousness was a predictor rather than a product of the social gradient.

The level of significance for all analyses was set at α=0.05. All statistical analyses were carried out using the Statistical Package for the Social Sciences, V.21.0 for Windows.^40^

## Results

Descriptive statistics for all variables included in the analysis are presented in table 1, separately for smokers and non-smokers. Differences between smokers and non-smokers were tested with χ^2^ tests for categorical and t tests for continuous variables. Smokers differed significantly from non-smokers on all variables except sex. Given the absence of sex differences, men and women were combined for all analyses.

Bivariate correlations (two-tailed Spearman for ordinal and two-tailed Pearson for continuous variables) between variables are presented in table 2. All variables besides sex and attention problems were significantly associated with each other. Importantly, childhood conscientiousness was significantly but only moderately associated with adulthood conscientiousness (r=0.15, p<0.01).

**Table 2:** Bivariate correlations (N=8218)

Variables	Sex (1=M; 2=F)	Social class at birth	Cognitive ability at 7	Attention problems at 7	Conduct problems at 7	Childhood conscientiousness	Social class at 50	Qualifications at 50	Conscientiousness at 50
Social class at birth (1=highest; 6=lowest)	0.01								
Cognitive ability at 7 (1=lowest; 5=highest)	0.03*	−0.12**							
Attention problems at 7 (1=lowest; 3=highest)	−0.04**	0.06**	−0.10**						
Conduct problems at 7	−0.18**	0.10**	−0.16**	0.15**					
Childhood conscientiousness	0.11**	−0.13**	0.10**	−0.09**	−0.19**				
Social class at 50 (1=highest; 6=lowest)	<0.01	0.21**	−0.17**	0.06**	0.15**	−0.20**			
Qualifications at 50 (0=lowest; 5=highest)	−0.03*	−0.24**	0.20**	−0.09**	−0.20**	0.30**	−0.46**		
Conscientiousness at 50	0.10**	−0.05**	0.05**	−0.01	−0.09**	0.15**	−0.10**	0.08**	
Smoking at 50 (0=No; 1=Yes)	<0.01	0.10**	−0.05**	0.04**	0.09**	−0.21**	0.15**	−0.16**	−0.05**

All significant correlations are in bold.

*p<0.05; **p<0.01; #p<0.10.

**Figure 1:**
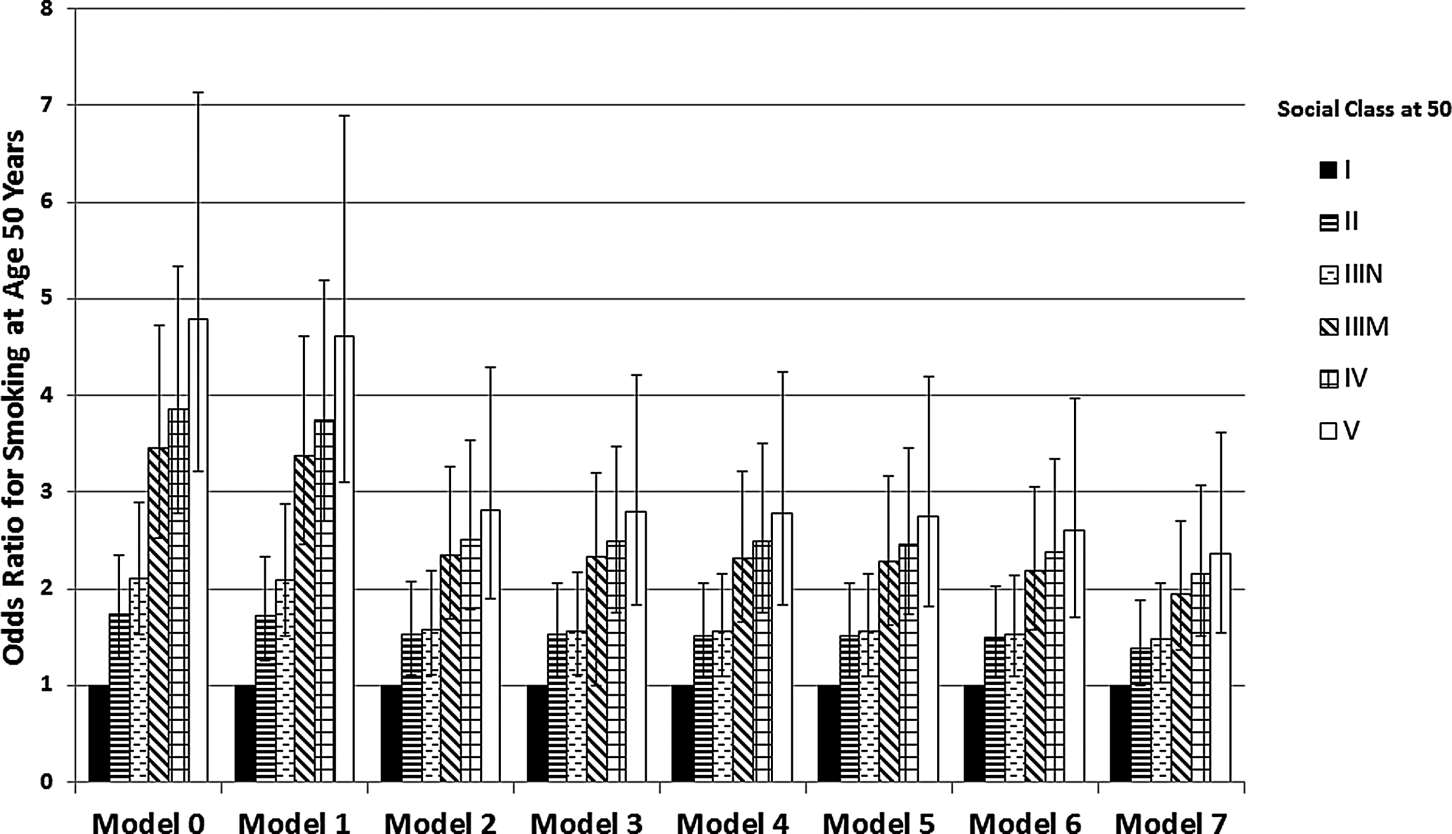
Variables in models: model 0: sex; model 1: sex, conscientiousness at 50; model 2: sex, conscientiousness at 50, qualifications at 50; model 3: sex, conscientiousness at 50, qualifications at 50, cognitive ability at 7; model 4: sex, conscientiousness at 50, qualifications at 50, cognitive ability at 7, attention problems at 7; model 5: sex, conscientiousness at 50, qualifications at 50, cognitive ability at 7, attention problems at 7, conduct problems at 7; model 6: sex, conscientiousness at 50, qualifications at 50, cognitive ability at 7, attention problems at 7, conduct problems at 7, social class at birth; model 7: sex, conscientiousness at 50, qualifications at 50, cognitive ability at 7, attention problems at 7, conduct problems at 7, social class at birth, childhood conscientiousness.

Results of the nested logistic models are shown in table 3 (changes in the social gradient of smoking for models 0–7 are also illustrated in figure 1). The row immediately beneath each variable gives the χ^2^ and degrees of freedom (DF). A significant change in χ^2^ allowing for the change in DF shows whether each additional variable significantly improved on the previous model. The first column of table 3 shows the association of smoking to social class adjusted for sex only (model 0). Smoking is, as expected, strongly related to adult social class, with those in the least advantaged class V at age 50 almost five times (OR=4.79, CI 3.21 to 7.14) more likely to smoke than those in the most advantaged class I. Adding adult conscientiousness to social class at age 50 produced a significantly better model (model 1) than social class and sex alone (χ^2^ improvement=10.9 with 1 added DF) with higher conscientiousness predicting lower likelihood of being a smoker at age 50 (OR=0.98, CI 0.97 to 0.995). However, the gradient in smoking between social class I and V was reduced by only 3.6%, indicating that the effects of social class and conscientiousness are largely independent. Inclusion of educational qualifications in model 2 greatly improved the fit of the total model, accounting for 41.1% of the social class difference in smoking (compared with model 0). The addition of cognitive ability at 7 in model 3 did not improve the model over and above the other variables explaining only an additional 0.7% of the social gradient compared with model 2. Similarly, model 4 which included attention problems at 7 did not lead to an improvement of the model and explained just an additional 0.2% of the social gradient compared with model 3. Inclusion of childhood conduct problems at 7 led to a significantly better fit, but explained only an additional 0.6% of the social gradient in smoking controlling for all other variables. Introduction of childhood social class in model 6 significantly increased explanatory power over model 5, reducing the social gradient of smoking by a total of 45.5% (compared with model 0). Finally, the addition of childhood conscientiousness in model 7 greatly improved the fit of the total model (χ^2^ increased 197.8 points for the addition of only one DF), with higher childhood conscientiousness predicting lower likelihood of being a smoker at age 50 (OR=0.86, CI 0.84 to 0.88), and further reduced the adult social gradient of smoking at age 50 (by 5%). Importantly, adult conscientiousness was no longer a significant predictor of smoking after inclusion of childhood conscientiousness. All variables combined in model 5 explained 50.5% of the difference in smoking between the most and least advantaged social classes at age 50 (model 0).

**Table 3:** Results of nested logistic models in the prediction of smoking status at 50 years (N=8218)

	Model 0: sex, social class at 50 OR (95% CI)	Model 1: model 0+adult conscientiousness OR (95% CI)	Model 2: model 1+educational qualifications OR (95% CI)	Model 3: model 2+cognitive ability at 7 OR (95% CI)	Model 4: model 3+attention problems at 7 OR (95% CI)	Model 5: model 4+conduct problems at 7 OR (95% CI)	Model 6: model 5+social class at birth OR (95% CI)	Model 7: model 6+childhood conscientiousness OR (95% CI)
Sex								
Male	1	1	1	1	1	1	1	1
Female	1.09 (0.97, 1.23)	1.11 (0.99, 1.25)#	1.10 (0.97, 1.24)	1.10 (0.97, 1.24)	1.10 (0.98, 1.25)	1.15 (1.01, 1.30)*	1.13 (1.00, 1.28)*	1.20 (1.06, 1.36)**
Social class at 50								
I	1	1	1	1	1	1	1	1
II	1.73 (1.27, 2.35)***	1.71 (1.26, 2.33)***	1.51 (1.11, 2.07)**	1.51 (1.10, 2.06)*	1.50 (1.10, 2.06)*	1.50 (1.10, 2.06)*	1.48 (1.08, 2.03)*	1.37 (0.99, 1.88)#
IIIN	2.10 (1.52, 2.90)***	2.08 (1.51, 2.87)***	1.56 (1.11, 2.18)*	1.55 (1.11, 2.17)*	1.54 (1.10, 2.16)*	1.54 (1.10, 2.16)*	1.52 (1.09, 2.14)*	1.46 (1.03, 2.06)*
IIIM	3.45 (2.52, 4.72)***	3.37 (2.46, 4.61)***	2.34 (1.69, 3.26)***	2.32 (1.67, 3.23)***	2.31 (1.66, 3.22)***	2.27 (1.63, 3.17)***	2.18 (1.57, 3.05)***	1.93 (1.37, 2.70)***
IV	3.85 (2.78, 5.34)***	3.74 (2.70, 5.19)***	2.50 (1.78, 3.53)***	2.48 (1.76, 3.51)***	2.48 (1.75, 3.50)***	2.45 (1.73, 3.46)***	2.36 (1.67, 3.34)***	2.15 (1.51, 3.07)***
V	4.79 (3.21, 7.14)***	4.62 (3.10, 6.89)***	2.82 (1.89, 4.29)***	2.79 (1.84, 4.25)***	2.78 (1.83, 4.24)***	2.75 (1.81, 4.19)***	2.61 (1.71, 3.97)***	2.37 (1.55, 3.62)***
X2 (DF)	200.11 (6)***							
Conscientiousness at 50		0.98 (0.97, 0.995)**	0.99 (0.98, 0.998)*	0.99 (0.98, 0.998)*	0.99 (0.98, 0.998)*	0.99 (0.98, 0.999)*	0.99 (0.98, 1.00)*	1.00 (0.98, 1.01)
X2 (DF)		211.01 (7)***						
		Model improvement: p<0.001						
Qualifications at 50								
1			1	1	1	1	1	1
2			0.72 (0.57, 0.89)**	0.72 (0.57, 0.90)**	0.72 (0.58, 0.90)**	0.73 (0.58, 0.92)**	0.74 (0.59, 0.93)**	0.80 (0.63, 1.00)*
3			0.55 (0.45, 0.66)***	0.55 (0.45, 0.67)***	0.55 (0.45, 0.67)***	0.58 (0.48, 0.70)***	0.59 (0.49, 0.72)***	0.70 (0.57, 0.85)**
4			0.47 (0.38, 0.59)***	0.48 (0.38, 0.59)***	0.48 (0.39, 0.60)***	0.50 (0.41, 0.63)***	0.52 (0.42, 0.64)***	0.60 (0.42, 0.85)**
5			0.38 (0.31, 0.46)***	0.38 (0.31, 0.47)***	0.39 (0.31, 0.47)***	0.41 (0.33, 0.50)***	0.43 (0.35, 0.53)***	0.59 (0.47, 0.74)***
6			0.31 (0.20, 0.48)***	0.32 (0.21, 0.49)***	0.32 (0.21, 0.49)***	0.35 (0.22, 0.53)***	0.37 (0.24, 0.56)***	0.80 (0.29, 0.83)**
X2 (DF)			317.39 (12)***					
			Model improvement: p<0.001					
Cognitive ability at 7								
Lowest				1	1	1	1	1
2nd				1.07 (0.90, 1.28)	1.07 (0.90, 1.28)	1.08 (0.91, 1.29)	1.08 (0.91, 1.29)	1.07 (0.90, 1.28)
3rd				0.97 (0.79, 1.18)	0.97 (0.80, 1.19)	0.99 (0.81, 1.21)	1.00 (0.81, 1.23)	0.98 (0.80, 1.20)
4th				1.04 (0.83, 1.22)	1.01 (0.83, 1.23)	1.04 (0.85, 1.27)	1.05 (0.86, 1.29)	1.04 (0.86, 1.27)
Highest				0.94 (0.77, 1.13)	0.95 (0.78, 1.14)	0.98 (0.81, 1.27)	1.00 (0.82, 1.21)	1.00 (0.83, 1.21)
X2 (DF)				320.33 (16)***				
				Model improvement: p=0.58				
Attention problems at 7								
Never					1	1	1	1
Sometimes					1.10 (0.95, 1.27)	1.08 (0.93, 1.24)	1.07 (0.93, 1.24)	1.03 (0.89, 1.19)
Frequently					1.15 (0.92, 1.43)	1.10 (0.88, 1.37)	1.08 (0.87, 1.35)	1.04 (0.83, 1.31)
X2 (DF)					323.51 (18)***			
					Model improvement: p=0.23			
Conduct problems at 7						1.05 (1.03, 1.08)***	1.05 (1.02, 1.08)***	1.03 (1.00, 1.06)*
X2 (DF)						339.30 (19)***		
						Model improvement: p<0.001		
Social class at birth								
I							1	1
II							1.00 (0.71, 1.42)	1.11 (0.65, 1.88)
IIIN							1.10 (0.77, 1.58)	1.08 (0.52, 2.22)
IIIM							1.20 (0.88, 1.65)	1.26 (0.76, 2.08)
IV							1.20 (0.84, 1.70)	1.22 (0.72, 2.06)
V/no job/single parent							1.74 (1.24, 2.44)**	1.75 (1.03, 2.97)*
X2 (DF)							370.58 (24)***	
							Model improvement: p<0.001	
Childhood conscientiousness								0.86 (0.84, 0.88)***
X2 (DF)								568.38 (25)***
								Model improvement: p<0.001
Explained proportion of social gradient in relation to model 0 (reduction)		3.6%	41.1%	41.8%	42.0%	42.6%	45.5%	50.5%

Nested logistic regression models were compared to investigate the influence of conscientiousness on the social gradient of smoking at age 50. The uncorrected social gradient for smoking based on social class at age 50 was tested in model 0 (controlled for sex). Models 1–7 then successively introduced adult conscientiousness, educational qualifications at 50, cognitive ability at age 7, attention problems at age 7, conduct problems at age 7, social class at birth and childhood conscientiousness at 16. The row immediately beneath each variable gives the χ^2^ and degrees of freedom (DF). A significant change in χ^2^ allowing for the change in DF shows whether each additional variable significantly improved the previous model. The explained proportion of social gradient in row 4 was calculated by relating the absolute difference between the OR of age 50 social class V of models 1–7 and the baseline OR of age 50 social class V in model 0 to the baseline OR (in (%)). *p<0.05; **p<0.01; ***p<0.001; #p<0.10.

In order to compare the effect of childhood conscientiousness on the social gradient of smoking before inclusion of the covariates, we ran an additional model similar to model 1 but with childhood conscientiousness rather than adult conscientiousness. Controlling only for sex, childhood conscientiousness was associated with less smoking at 50 years (OR=0.85, CI 0.82 to 0.87) and significantly reduced the association between smoking and social class by 28.0%.

## Discussion

Consistent with previous investigations, higher conscientiousness scores in childhood and adulthood were found to predict lower likelihood of smoking at age 50. Importantly, before inclusion of important covariates, 3.6% of the social gradient in smoking at age 50 could be attributed to differences in adult conscientiousness (and 28.0% to childhood conscientiousness). Adult conscientiousness remained a significant predictor of smoking behaviour at age 50 even after controlling for educational attainment at age 50, cognitive ability, attention and conduct problems at age 7 and social class at birth, suggesting that the association between adult conscientiousness and smoking was not simply a function of continuities in social advantage and cognitive ability. Based on the ORs of the final model, model 7, educational attainment by age 50 was the strongest predictor of smoking behaviour—besides social class at 50—also explaining the largest proportion of the social gradient of smoking. Differences in childhood cognitive ability and attention problems were less strongly associated with smoking behaviour at 50 and did not remain significant after controlling for educational qualifications in adulthood. Childhood conduct problems were a significant predictor of adulthood smoking but largely independent of childhood conscientiousness.^39^ Childhood social class emerged as a further significant predictor of smoking behaviour at age 50, explaining 2.9% of the social gradient in smoking independent of social class at 50, cognitive ability, attention and conduct problems in childhood and educational qualifications at 50—a finding consistent with other studies.^41^ ^42^ Finally, the effect of childhood conscientiousness on adult smoking was not only highly significant but also explained an additional 5.0% of the social gradient independent of all other variables. Childhood conscientiousness also reduced the estimate for conscientiousness measured in adulthood to non-significance, suggesting that protective effects of adult conscientiousness may, in fact, arise during childhood. That childhood conscientiousness explained a small proportion of the social gradient of smoking at age 50—independent of educational attainment, childhood cognitive ability, attention and conduct problems and childhood social class—suggests that childhood conscientiousness is not merely an indicator of social class or cognitive ability.

The strengths of the study include the longitudinal data over 50 years of the life course, and the availability of contemporaneous measures of childhood psychological characteristics measured long before the Big Five personality constructs had been developed, but able to be matched to these. Further strengths are the large sample size and the fact that no measures were dependent on retrospective recall by study participants. However, the findings of this analysis have to be considered in light of several limitations: (1) the study is based on a correlational design which does not allow for causal interpretation; (2) the sample suffered substantial attrition over time with smokers and those of lower social status and lower conscientiousness scores less likely to be included at the 50-year assessment; (3) the item from which the dependent variable smoking was derived did not ask specifically about the smoking of cigarettes, as distinct from cigars, pipes, etc; (4) several of the covariates have been assessed with only a few or single items (eg, attentional and conduct problems) and (5) other potentially important covariates have not been included (eg, parental smoking, smoking-related medical issues).

However, this study builds on the growing literature investigating the relationship between personality traits and health behaviours, overcoming significant limitations of existing work by taking a life course perspective in a large cohort study. The current work has made use of a ‘Big Five’ personality questionnaire administered at age 50 in the National Child Development Study but, in addition, included a childhood conscientiousness proxy measure based on three cohort-member-rated items assessed by questionnaires at 16 years. Importantly, individual differences in conscientiousness explained only a small proportion of the social gradient of smoking at age 50 after taking account of childhood social class, cognitive ability, attention and conduct problems as well as educational attainment. In fact, all included variables in total accounted for slightly more than 50% of the social gradient of smoking. Nevertheless, inclusion of the personality measures added a small but significant component to the understanding of health inequalities. The current findings propose that research on health inequalities may benefit from including the notion of personality traits and investigating their effects on health behaviours across life.

The observation that childhood conscientiousness rather than adulthood conscientiousness predicted smoking behaviour at age 50 may suggest that preventative efforts as well as clinical interventions aimed at decreasing health-risk behaviours will benefit from specifically targeting personality traits associated with health behaviours––in childhood rather than adulthood. Empirical evidence that such personality-targeted interventions are effective emerged recently regarding the reduction of alcohol use in adolescents.^43^

An important question for future research will be the investigation of specific mechanisms involved in the association between high conscientiousness in childhood with lower likelihood of smoking in adulthood. Recently, self-control in childhood has been identified as one important factor for health outcomes in adulthood^26^ ^39^ but there are likely to be other mechanisms involved in the protective effects of conscientiousness (eg, compliance with treatment and preventive advice).

In conclusion, using longitudinal prospective data covering 50 years over the life course in a large sample from a representative cohort study, childhood conscientiousness emerged as a significant predictor of smoking at age 50, explaining 5.0% of the social gradient of smoking, independent of adult conscientiousness, educational qualifications in adulthood, childhood cognitive function, childhood attention and conduct problems and childhood social class. Importantly, childhood conscientiousness emerged as a stronger predictor of smoking than adult conscientiousness and statistically accounted for the association of conscientiousness measured at age 50 with smoking, suggesting that the personality trait conscientiousness is a predictor rather than a product of social differences in smoking.

**What is already known on this subject**

- Most health outcomes are unequally distributed across the different socioeconomic strata with those from more disadvantaged backgrounds reporting more health problems.
- Health behaviours, including smoking, are distributed similarly across the different socioeconomic groups and are hypothesised to play a major role in accounting for the social gradient of health outcomes.
- Recently, empirical studies identified the personality trait conscientiousness as an additional important predictor of health outcomes, health behaviours and the social gradient associated with both. However, most existing studies are cross-sectional and do not allow testing whether conscientiousness is simply a marker of social inequalities or rather a predictor of the social gradient across the life course.

**What this study adds**

- Testing in a large cohort study whether conscientiousness predicts smoking and its social gradient at 50 years, it was found that higher conscientiousness in childhood and adulthood both predicted lower likelyhood of smoking at 50 years accounting for a small but significant proportion of the social gradient of smoking independent of other factors.
- Applying a life course approach, it was childhood conscientiousness rather than adulthood conscientiousness that was most strongly associated with smoking at 50 years, suggesting that conscientiousness is a predictor of adult smoking behaviour rather than a result of exposure to social inequality across life.
- Findings of this study suggest that preventative efforts related to health-risk behaviours would benefit from early interventions targeting personality traits associated with health behaviours.
